# *microRNA‐126* inhibits vascular cell adhesion molecule‐1 and interleukin‐1beta in human dental pulp cells

**DOI:** 10.1002/jcla.24371

**Published:** 2022-03-25

**Authors:** Long Jiang, Tadkamol Krongbaramee, Xinhai Lin, Min Zhu, Yaqin Zhu, Liu Hong

**Affiliations:** ^1^ Department of General Dentistry Shanghai Ninth People’s Hospital Shanghai Jiao Tong University School of Medicine College of Stomatology Shanghai Jiao Tong University National Center for Stomatology National Clinical Research Center for Oral Diseases Shanghai Key Laboratory of Stomatology Shanghai China; ^2^ 4083 Iowa Institute for Oral Health Research College of Dentistry The University of Iowa Iowa City Iowa USA

**Keywords:** human dental pulp cells, inflammation, interleukin‐1β, *microRNA‐126*, vascular cell adhesion molecule‐1

## Abstract

**Background:**

Vascular cell adhesion molecule (VCAM‐1) mediates pulpitis via regulating interleukin (IL)‐1β. microRNA (*miR)*‐*126* was reported to regulate the VCAM‐1 under many different pathophysiological circumstances. We investigated variations of *miR*‐*126* and VCAM‐1 in inflamed patient pulp tissues and determined potential roles of *miR*‐*126* in pulpitis using human dental pulp cells (hDPCs) in vitro.

**Methods:**

We quantitatively measured the transcripts of *miR*‐*126* and VCAM‐1 in inflamed human pulp tissues using qRT‐PCR and compared with those from healthy human pulp tissues. In addition, we transfected *miR*‐*126* in hDPCs using plasmid DNA (pDNA)‐encoding *miR*‐*126* delivered by polyethylenimine (PEI) nanoparticles.

**Results:**

The irreversible pulpitis significantly reduced *miR*‐*126* and increased the transcript of VCAM‐1 in pulp tissues (*p* < 0.05). pDNA‐encoding *miR*‐*126* delivered PEI nanoparticles and effectively upregulated the expression of *miR*‐*126* in hDPCs (*p* < 0.05). The overexpression of *miR*‐*126* could effectively suppress the transcripts and protein levels of VCAM‐1 and IL‐1β induced by Pg‐LPS at 100ng/mL in DPCs (*p* < 0.05).

**Conclusions:**

*miR*‐*126* is involved in pulpitis and downregulated the VCAM‐1 and IL‐1β in DPCs. *miR*‐*126* may be a potential target to attenuate the inflammation of pulpitis.

## Graphical abstract

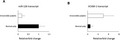

## 1 INTRODUCTION

Pulpitis is a kind of progressive inflammation in the dentin–pulp complex caused by deep caries lesions, trauma, or preparation techniques for removing caries lesions.^1^ Increased internal pressure in the pulp chamber by the bacterial infection and subsequent inflammation causes pulp tissue ischemia and severe pain. The inflammation generally is a protective defense response to infection and injury in the body^2^ by removing the infection and promoting wound healing and tissue restoration.^3^ However, the inflammation of pulpitis is a double‐edged sword for healing pulpal tissues. It has been demonstrated that a relatively low amount of inflammation promotes dentin repair, whereas a high amount of chronic inflammation may inhibit repair mechanisms.^4^, ^5^ Thus, modulating the inflammatory reaction plays an important role in treating pulpitis and accelerating dentinogenesis. Inflammation involves inflammatory cell activation, cellular factor secretion, mediation of antigen‐antibody reaction, accompanying increased blood flow, and dilation of venules and arterioles, enhancement of blood vessels permeability, and percolation of leukocytes into the tissues.^6^, ^7^ The vascular cell adhesion molecule‐1 (VCAM‐1/CD106) (a 90‐kDa glycoprotein), a member of cell adhesion molecules (CAMS), plays a crucial role in accumulating inflammatory cells during inflammation by regulating the adhesion of lymphocytes monocytes, eosinophils, and basophils to vascular endothelium.^8^, ^9^, ^10^, ^11^, ^12^ VCAM‐1 was reported to actively involve the inflammation of pulpitis in tooth preparation. Lipopolysaccharide (LPS) increased VCAM‐1 in human dental pulp cells (DPCs).^13^, ^14^ These evidence indicate that VCAM‐1 may contribute to the excessive inflammation in pulpitis,^15^ and thus, the modulation of VCAM‐1 may effectively attenuate the inflammatory reaction of pulpitis.

MicroRNAs (miRs) are short non‐coding RNAs that regulate physiologically and pathophysiologically through translational inhibition or degeneration of specific genes’ mRNAs.^16^ Growing evidence indicates that miRs play essential roles in pulpal inflammation and pulpal tissue repair. They may be used for pulpitis treatment.^17^, ^18^ *miR*‐*126* (also referred to as *miR*‐*126*‐*3p*) is derived from the egfl7 gene, harboring within intron 7 in all vertebrates.^19^ *miR*‐*126* serves as a crucial regulator in endothelial cell functions, including vascular repair, angiogenesis, inflammatory activation, and apoptosis. Specifically, *miR*‐*126* represses SPRED1 and PIK2R2, which negatively regulate VEGF signaling via the MAPK/ERK and PI3K/AKT pathways, respectively, to promote the cells proliferation, migration, and angiogenesis.^19^ *miR*‐*126* could also enhance the maturation and stabilization of growing blood vessels by suppressing the p21‐activated kinase 1 gene and regulating angiopoietin‐1 signaling.^20^ Furthermore, *miR*‐*126* was reported to modulate the inflammation through directly targeting VCAM‐1 to block the adhesion and infiltration of leukocytes into the vasculature wall.^19^, ^21^ However, the characteristics of *miR*‐*126* in the dental pulp and its regulatory roles in pulpal inflammation remain unknown.

In this study, we investigated the *miR*‐*126* and VCAM‐1 variation in inflamed pulp tissues and determined the inhibitory function of *miR*‐*126* in VCAM‐1 and pulpitis using human dental pulp cells (DPCs).

## 2 MATERIALS AND METHODS

### 2.1 Collection of dental pulp tissue

This study was approved by the Institutional Review Boards of the University. Each patient signed written informed consent. Healthy dental pulp was obtained from 10 patients with an average age of 19.25 ± 3.3 years old from the extracted wisdom teeth or premolars of patients whose teeth were removed for orthodontic reasons. The inflamed pulp tissues were extirpated from carious teeth of 10 patients with an average of 25.5 ± 11.6 years old diagnosed with irreversible pulpitis according to the American Association of Endodontists guidelines.^22^ The dental pulp was exposed with a handpiece and extirpated with a nerve broach.

### 2.2 Cell culture

Human DPCs were isolated and cultured as described previously.^23^ Briefly, the pulp tissue was carefully stripped from the crown and root and cut into pieces smaller than 1mm.^3^ The tissue fragments were then covered by the glass coverslips on the bottom of culture dishes and incubated with DMEM (Gibco) supplemented with 100 IU/ml penicillin (Gibco) and 20% fetal bovine serum (FBS, Gibco). The culture medium was changed at five‐day intervals. After reaching 70% confluence, the cells were collected by trypsinization (0.2% trypsin and 0.02% EDTA, Gibco) and split at a ratio of 1:4 and subcultured with DMEM supplemented with 10% FBS.

### 2.3 Transfection of DPCs with plasmid DNA (pDNA) encoding *miR‐126*

The pDNA‐encoding *miR*‐*126* was constructed with pSilencer‐4.1 and the *miR*‐*126*‐specific sequence commercially (OriGene). pSilencer‐4.1 was used as a vector stably expressing *miR*‐*126*, and the mature sequence of *miR*‐*126* (CAUUAUUACUUUUGGUACGCG) was synthesized by oligonucleotides. Empty pSilencer‐4.1 vector (EV) was used as a control. Polyethyleneimine (PEI) was used to facilitate the transfection of plasmid DNA (pDNA)‐encoding miR‐126 as described in our previous studies.^24^ Briefly, pDNA‐encoding *miR*‐*126* and PEI at a ratio of 1:3 were mixed in an opti‐MEM medium (Gibco) for 30 s and incubated for 20 min at room temperature to form nanoplexes. A total of 500 μl of opti‐MEM containing 3 µg PEI and 9 µg pDNA of *miR*‐*126* was added to DPCs at 5 × 10^5^ cells/well in a 6‐well plate. After 4 h, DPCs were washed twice using PBS and then cultured with DMEM for 48 h. The overexpression of *miR*‐*126* was measured using qRT‐PCR.

### 2.4 Influence of *miR‐126* on VCAM‐1 and IL‐1β under LPS challenge

After transfection with the pDNA‐encoding *miR*‐*126* at 9µg pDNA delivered by PEI in a 6‐well plate for 48 h, the DPCs were treated using Pg‐LPS (Sigma‐Aldrich) at 100 ng/ml for 6 and 24 h. The expression of *miR*‐*126* and transcripts of *VCAM*‐*1* and *IL*‐*1β* were analyzed by qRT‐PCR. The protein level of VCAM‐1 was measured by Western blot using the polyclonal antibody against human VCAM‐1 (1:1000, Abcam) 24 h after Pg‐LPS challenge. IL‐1β in the supernatant was quantified using an ELISA kit (Neobioscience) according to the manufacturer's protocol.

### 2.5 qRT‐PCR

Total RNA from human pulp tissue and cultured cells were collected using an miReasy mini kit (Qiagen). The concentration and purity of the total RNA were quantified using a NanoDrop™ One Microvolume UV‐Vis Spectrophotometer (Thermo Fisher Scientific). The measurement of *miR*‐*126* expression was performed using the mirScript reverser transcription kit and miRScript SYBR Green PCR Kit (Qiagen). mRNA expression of VCAM‐1 and IL‐1β was measured by qRT‐PCR using PrimeScript ™ Reagent Kit (Takara) to carry out reverse transcription and amplified reaction by using amplification primers with SYBR Green PCR Master Mix (Takara). The comparative ΔΔCt method was used to quantify the relative level of different mRNA expression. All samples were normalized to GAPDH. The probers for *miR*‐*126* were designed and synthesized by the Qiagen company. The PCR primers specific for GAPDH were 5′‐CTGGGCTACACTGAGCACC‐3′ (Forward) and 5′‐AAGTGGTCGTTGAGGGCAATG‐3′ (Reverse); VCAM‐1:5′‐TTTGACAGGCTGGAGATAGACT‐3′ (Forward) and 5′‐TCAATGTG TAATTTAGCTCGGCA‐3′ (Reverse); IL‐1β: 5′‐TTCGACACATGGGATAACGAGG‐3′ (Forward) and 5′TTTTTGCTGTGAGTCCCGGAG‐3′ (Reverse).

### 2.6 Statistical analysis

The data were analyzed with commercially available statistics software (Statistical Analysis System 8.2). All quantitative results were expressed as mean ± standard deviation. The differences in relatively normalized expression for *miR*‐*126 and* VCAM‐1 between health and inflamed pulp were determined using Student's *t* test. Statistical significance among groups with *miR*‐*126* treatment was determined using a one‐way analysis of variance (ANOVA), and the Bonferroni post hoc test was used for multiple comparisons. A *p*‐value of < 0.05 was considered to be significant. Each experiment was performed in triplicate.

## 3 RESULTS

### 3.1 Pulpal inflammation reduced *miR‐126* and increased VCAM‐1

**FIGURE 1:**
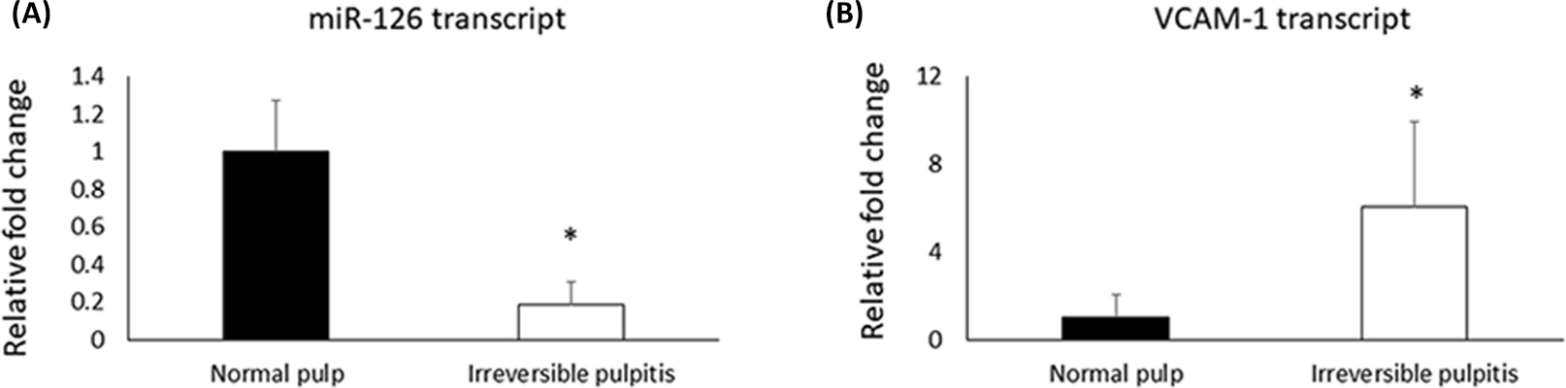
Irreversible pulpitis downregulated *miR*‐*126* and increased VCAM‐1 in human pulpal tissues. *p < 0.05, *n =* 6

Pulpal tissues were collected from patients diagnosed with irreversible pulpitis and healthy pulpal tissue as controls. *miR*‐*126* was significantly decreased in inflamed pulp tissues (Figure 1A), while the transcript of VCAM‐1 was significantly increased (*p* < 0.05) (Figure 1B).

### 3.2 PEI facilitated the transfection of *miR‐126* into human DPCs

**FIGURE 2:**
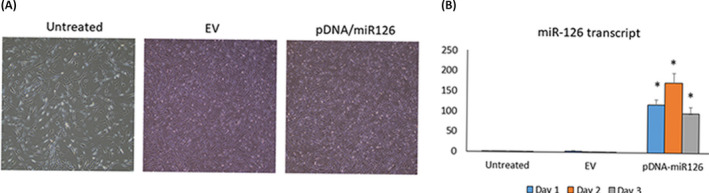
Polyethylenimine nanoparticles effectively transfected pDNA‐encoding miR‐126 into DPCs. (A) Microphotographs of DPCs transfected with pDNA‐encoding *miR*‐*126* or EV (100×); (B) the fold change in *miR*‐*126* in DPCs after transfection for 1, 3, and 7 days. *p < 0.05 versus Untreated

We used PEI nanoparticles to deliver pDNA‐encoding *miR*‐*126* to primary human DPCs collected from 12 different patients (6 male and 6 female). After treating with 3 μg pSil‐miR‐126 or EV in 600 μl opti‐MEM, DPCs maintain the fibroblast‐like morphology (Figure 2A). For DPCs treated with *miR*‐*126*, *miR*‐*126* expression was significantly increased than that with EV after 24 h. The expression level of *miR*‐*126* maintains a high level after 7 days (*p* < 0.05) (Figure 2B).

### 3.3 LPS inhibited *miR‐126* and upregulated the expression of VCAM‐1 and IL‐1β in DPCs

**FIGURE 3:**
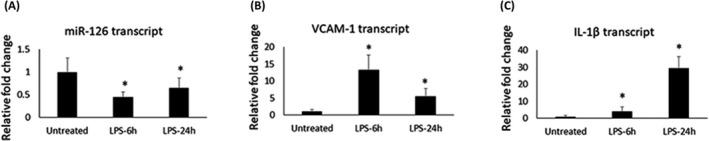
Lipopolysaccharide reduced *miR*‐*126* expression and increased VCAM‐1 and IL‐1β in human DPCs. A‐C. Normalized transcripts of *miR*‐*126* (A), VCAM‐1 (B), and IL‐1β in DPCs after treatment with LPS at 100 ng/ml. *p < 0.05 versus Untreated. Performed in triplicate

Dental pulp cells were stimulated with 100 ng/ml LPS for 6 or 24 h. LPS significantly reduced *miR*‐*126* expression after 6 and 24 h (*p* < 0.05) (Figure 3A). Meanwhile, the transcripts of VCAM‐1 and IL‐1β were significantly increased (*p* < 0.05) (Figure 3B,C). The level of VCAM‐1 decreased gradually after 24 h, while IL‐1β continuously increased.

### 3.4 Overexpression of *miR‐126* attenuated the IL‐1β and VCAM‐1

**FIGURE 4:**
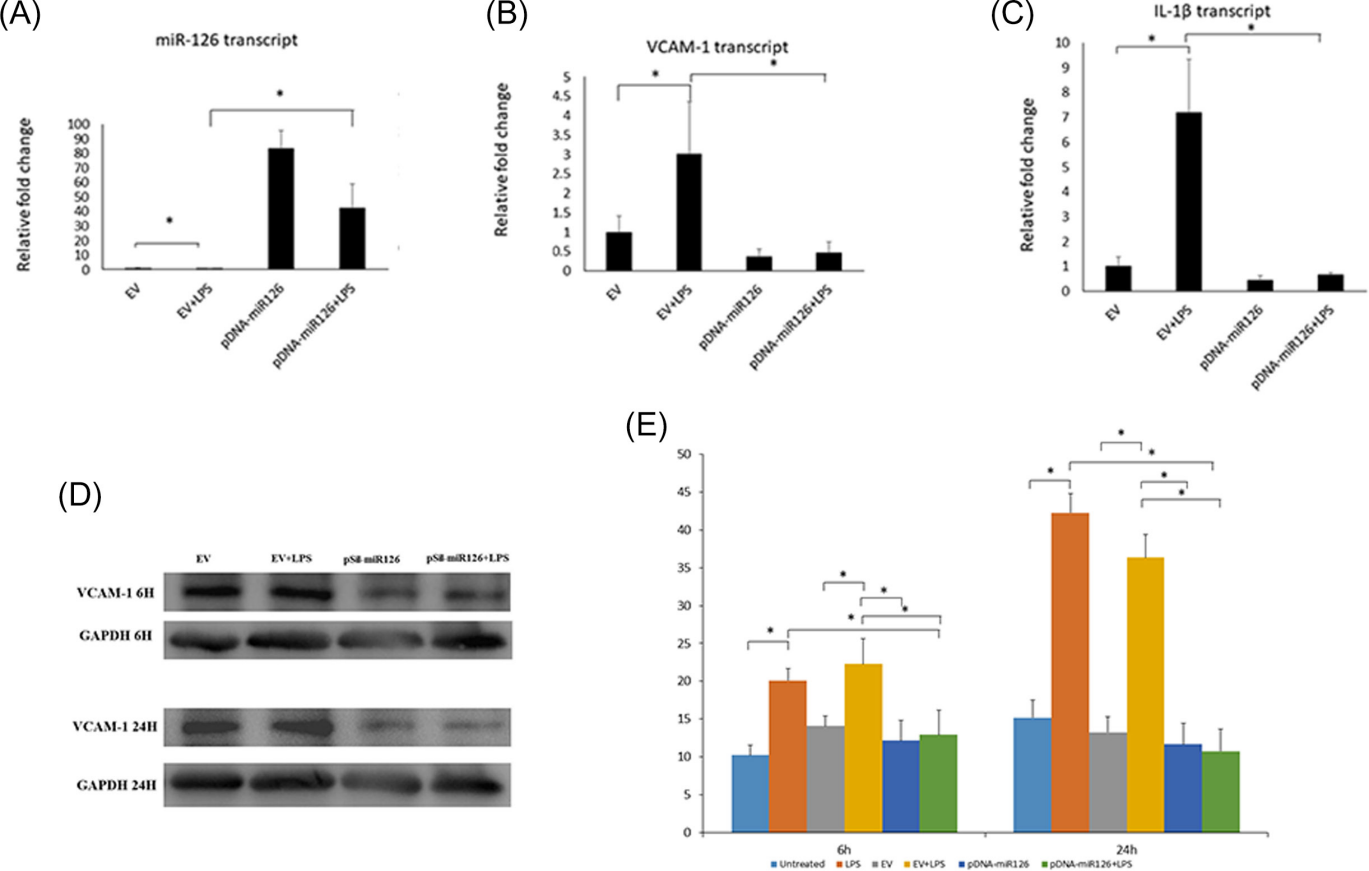
Overexpression of *miR*‐*126* inhibited VCAM‐1 and IL‐1β in DPCs. A–C. Normalized transcripts of *miR*‐*126* (A), VCAM‐1 (B), and IL‐1β in DPCs pretreated with pDNA‐encoding miR‐126 or EV after treatment with LPS at 100 ng/ml; D. Western blot of VCAM‐1 in DPCs pretreated with pDNA‐encoding *miR*‐*126* after PG‐LPS challenge; E. miR‐126 inhibited the protein level of IL‐1β measured by the ELISA in DPCs induced by PG‐LPS for 6 or 24 h. *p < 0.05. Performed in triplicate

The inhibitory function of *miR*‐*126* on VCAM‐1 and IL‐1β was investigated in DPCs with *miR*‐*126* overexpression. DPCs were transfected with pDNA‐encoding *miR*‐*126* or EV for 48 h and subsequently exposed to Pg‐LPS at 100 ng/ml. Transfection with *miR*‐*126* delivered by PEI nanoparticles significantly increased the expression of miR‐126 in DPCs (*p* < 0.05). While PG‐LPS reduced *miR*‐*126* in cells transfected with *miR*‐*126* and EV (Figure 4A), *miR*‐*126* was significantly increased in cells with *miR*‐*126* transfection than that with EV (*p* < 0.05) (Figure 4B). In addition, for cells induced by Pg‐LPS, *miR*‐*126* significantly reduced VCAM‐1 and IL‐1β than EV (*p* < 0.05). Pg‐LPS induced a slight increase in *VCAM*‐*1* or *IL*‐*1β* in the DPCs with overexpression of *miR*‐*126* (Figure 4B,C). In the Western bolt analysis, *miR*‐*126* overexpression reduced the expression of VCAM‐1 after the stimulation with Pg‐LPS than that with EV (Figure 4D). We measured the protein level of IL‐1β in the supernatant using ELISA. Pg‐LPS significantly increased the expression of IL‐1β protein in the DPCs alone or treated with EV (*p* < 0.05). However, no difference was observed in DPCs with overexpression of *miR*‐*126* (*p *> 0.05), indicating that pDNA‐encoding *miR*‐*126* effectively reduced IL‐1β level after Pg‐LPS challenge (Figure 4E).

## 4 DISCUSSION

In this study, we found that the expression of *miR*‐*126* in inflamed patient pulp tissues with increased VCAM‐1 was significantly downregulated compared with that of healthy pulp tissues. In addition, we confirmed the function of *miR*‐*126* overexpression in reducing VCAM‐1 and interleukin 1 beta (IL‐1β), a pro‐inflammatory cytokine participating in pulpitis, in human DPCs under the stimulation of LPS.

*miR*‐*126* has been reported to reduce IL‐6, IL‐10, and tumor necrosis factor‐α (TNF‐α) in endothelial cells via the PI3K/Akt/eNOS signaling pathway.^25^ It also directly targets VCAM‐1 and high mobility group box 1 (HMGB1), which are genes associated with endothelial activation and inflammation.^26^ In this study, we found that the inflamed pulpal tissues from irreversible pulpitis patients significantly decreased the *miR*‐*126* expression. The downregulation of *miR*‐*126* was associated with an increased VCAM‐1 in the inflamed pulpal tissues. Our studies also demonstrated that PEI nanoparticles effectively facilitated the transfection of pDNA‐encoding *miR*‐*126* to human DPCs. The overexpression of *miR*‐*126* delivered by PEI effectively downregulated VCAM‐1 and the IL‐1β, a key pro‐inflammatory cytokine in pulpitis, under LPS challenge in vitro. These results strongly indicated that *miR*‐*126* might be useful for pulpitis treatment and dentin regeneration by targeting VCAM‐1 and attenuating inflammation.

Bacterial endotoxin LPS is a key factor initiating the inflammation of pulpitis.^27^ LPS activates inflammatory cytokines, such as IL‐1, IL‐6, IL‐8, matrix metalloproteinase (MMP)‐9, MMP‐2, and TNF‐α^28^, ^29^, ^30^ in the pulpitis progress. In this study, we found that Pg‐LPS significantly reduced *miR*‐*126* and upregulated VCAM‐1 in human DPCs in vitro. These results supported our finding that there was downregulation of *miR*‐*126* and upregulation of VCAM‐1 in inflamed patient pulp tissues, although the mechanism of the LPS inhibition on the miR‐126 biogenesis is still not clear. Because *miR*‐*126* directly targets VCAM‐1, the reduced *miR*‐*126* by LPS may contribute at least partially to the upregulation of VCAM‐1 in pulpitis and DPCs in vitro. Like other pro‐inflammatory cytokines, VCAM‐1 has been demonstrated to play critical roles in inflammation in pulpitis by regulating inflammatory cell migration and binding on the endothelium surface.^31^ This finding suggested that the downregulated *miR*‐*126* might contribute to the inflammatory progression of pulpitis by activating VCAM‐1, whereas overexpression of *miR*‐*126* might attenuate the inflammation in pulpitis.

A practical and safe gene delivery system is the key to the development of miR‐based gene therapy for pulpitis.^32^ Considering the serious safety issues with viral vectors, non‐viral gene delivery systems are preferred for gene therapy. Among the currently reported non‐viral vectors, high molecular weight branched PEI is a gold standard and has been most widely used in preclinical studies and clinical trials due to its relatively high nucleic acid transfer efficiency and biocompatiblities.^33^ In this study, we used PEI nanoparticles to facilitate the transfection of plasmid encoding *miR*‐*126* into DPCs. No obvious toxicity of the transfection of pDNA‐encoding *miR*‐*126* delivered by PEI was found based on DPC morphology and IL‐1β measurement. The *miR*‐*126* delivered by PEI nanoparticles significantly upregulated the expression of miR‐126, and a high level of overexpression can last more than one week. In addition, overexpression of miR‐126 effectively downregulated VCAM‐1 and IL‐1β in DPCs. This evidence supported that PEI might serve as a non‐viral delivery system to deliver miR‐126.

Interleukin‐1β gene is a well‐known pro‐inflammatory cytokine in initiation and progression of inflammation, including macrophage recruitment, activation, and inducement of other pro‐inflammation cytokines, such as IL‐6, IL‐8, ICAM‐1, and modulating chemokine expression.^34^, ^35^, ^36^ IL‐1β was reported to be significantly increased in inflamed dental pulp tissue and DPCs under LPS stimulation.^37^ Inhibition of IL‐1β can relieve cell damage in inflammation,^38^, ^39^ and the imbalance between IL‐1β agonist and antagonist levels can lead to exaggerated inflammatory responses.^40^ It was reported that patients obtained beneficial effects from using IL‐1β antagonists.^41^ IL‐1β is not the direct target gene of *miR*‐*126*, and the regulation of *miR*‐*126* on IL‐1β is varied by cell types and inflammation. However, in this study, we found the *miR*‐*126* overexpression could significantly decrease IL‐1β in DPCs. These findings further supported the therapeutic potential of miR‐126 in pulpitis treatment. One previous study has demonstrated that *miR*‐*126* participated in the regulation of cell viability of DPCs by directly repressing PTEN levels and indirectly by activating Akt signal that can function as pro‐apoptotic factors.^42^ Our study has proposed different mechanisms of the effect of *miR*‐*126 on* DPCs.

In conclusion, in this study, we revealed *miR*‐*126* and VCAM‐1 variations in patients with irreversible pulpitis. LPS effectively downregulated *miR*‐*126* that might contribute to the pulpitis's inflammatory progression by activating VCAM‐1 and IL‐1β. Overexpression of *miR*‐*126* using non‐viral nanoparticle PEI can effectively suppress VCAM‐1 and reduce IL‐1β. These results indicated that *miR*‐*126* might be a potential target to treat pulpitis, and future studies were needed to confirm the regulation of *miR*‐*126* in vivo.

## CONFLICT OF INTEREST

The authors declare that they have no competing interest.

## CONSENT

Each patient signed written informed consent.

## Data Availability

All data generated or analyzed during this study are included in this. Further enquiries can be directed to the corresponding author.
